# Beyond Membrane Remodeling: Organelle Crosstalk and Convergent Pathology in Centronuclear Myopathy

**DOI:** 10.3390/muscles5020035

**Published:** 2026-05-08

**Authors:** Bana Abolibdeh, Charles H. Williams

**Affiliations:** 1 Department of Medicine, Michigan State University College of Human Medicine, East Lansing, MI 48824, USA; libdehba@msu.edu; 2 Henry Ford Health + Michigan State University Health Sciences, Detroit, MI 48202, USA

**Keywords:** centronuclear myopathy (CNM), Myotubularin 1 (*MTM1*), X-linked myotubular myopathy (XLMTM), Dynamin 2 (*DNM2*), reactive oxygen species (ROS), mitochondria, t-tubule/triad, cytoskeletal instability

## Abstract

Centronuclear myopathy (CNM) is a genetically heterogenous congenital myopathy traditionally classified as a membrane remodeling disorder. Emerging evidence reveals that centronuclear myopathy mutations converge upon common cellular dysfunction extending beyond membrane trafficking. This review proposes a unified model positioning CNM as a disorder of impaired organelle communication and structural crosstalk. We focus on how mutations in Myotubularin1 (*MTM1*) and gain-of-function mutations in Dynamin 2 (*DNM2*) disrupt the triad architecture, leading to aberrant calcium handling, mitochondrial dysfunction, imbalanced reactive oxygen species (ROS) production, and defective autophagy. These dysfunctions are not isolated but form a pathological feedback loop that compromises muscle integrity and regeneration. By identifying shared mechanisms across CNM types, this review positions the disorder as the convergence of organelle stress and cytoskeletal network failure. This perspective reveals novel therapeutic strategies based on the principle that targeting a central pathological node may alleviate systemic dysfunction. However, given the complexity of the organelle feedback loop, a comprehensive, multi-target approach may ultimately be required to achieve full phenotypic rescue across all affected tissues.

## 1. Introduction

Neuromuscular disease (NMD) represents a heterogenous group of disorders that impact motor neurons, peripheral nerves, neuromuscular junctions, and skeletal muscles. Mitochondrial dysfunction has emerged as a central factor in this disease, acting as a primary driver or a secondary contributor due to the high energy demands of muscle tissue. Disruption of mitochondrial function impairs ATP production and alters reactive oxygen species (ROS) levels. This can trigger endoplasmic reticulum (ER) stress at associated contact sites and membranes, exacerbate oxidative stress and inflammation, and ultimately lead to cell death [1]. Several NMDs, including centronuclear myopathies (CNM) and Charcot–Marie–Tooth (CMT) neuropathies, have been linked with mitochondrial dysfunction and membrane remodeling protein mutations of myotubularins, dynamins, and amphiphysins [2,3].

CNM is a rare, genetically heterogenous congenital NMD characterized by muscle weakness and the central localization of cellular nuclei in muscle fibers. Additional pathological features include structural defects in t-tubule organization and mitochondrial dysfunction, resulting in increased oxidative stress driven by genetic mutations. CNM disorders often present at birth or in early childhood, with an incidence of 1 in 50,000 births, and, although considered a progressive disease, cases progress slower when compared to myotonic dystrophies or metabolic myopathies [1,2,3,4].

CNM was first characterized as a case of myotubular myopathy in a 12-year-old patient presenting with muscle weakness, hypotonia, respiratory difficulties, and delayed motor function, consistent with congenital myopathies. A muscle biopsy revealed ~85% of skeletal muscle fibers presenting with centrally localized nuclei surrounded by a halo, as well as increased oxidative enzyme activity [5]. This study provided the first phenotypic description of the disease and the defining phenotype for the subsequent studies of conditions that would gain the moniker “centronuclear myopathy”. Laporte et al. identified the genetic basis of CNM by mapping mutations to the X chromosome. These mutations occur in the *MTM1* gene, which encodes the lipid phosphatase myotubularin. An analysis of 55 families revealed a variety of loss-of-function mutations of the *MTM1* gene linked to the CNM phenotype. This provided the first direct genetic link to the X-linked recessive subtype of CNM, X-linked myotubular myopathy (XLMTM) [6]. In a study conducted by Sher et al. [7], similar symptoms of muscle weakness and wasting were documented in a family with multiple affected members. Notably, the hereditary patterns showed that siblings were impacted, but not parents. Furthermore, there was no restriction of the disease to males and no clear maternal transmission pattern, which would be expected in an X-linked disease. This suggested a recessive inheritance pattern, adding to the genetic heterogeneity [7]. In a later study by Pavone et al., a familial pattern was observed where multiple family members were affected by CNM. The primary case was of a girl presenting with CNM symptoms. Family history revealed several members presenting with similar phenotypes who died at younger ages, suggesting a progressive familial condition. Contrary to Sher et al.’s observation, this familial dynamic presented an autosomal dominant mutation pattern, highlighting the variability that would later be confirmed in this disease [8].

Further clinical analyses followed, including Bethlem et al., highlighting the central nuclei observation along with type 1 fiber atrophy and myotube-like structures, further suggesting developmental arrest in the muscle maturation process. The central positioning of nuclei is consistent with immature myotube formation [9]. Collectively, these early studies established the foundation for characterizing CNM subtypes and their associated phenotypes, while revealing the first direct genetic links to the disease. Follow-up studies over the next few years identified additional genetic targets in the autosomal recessive inheritance that included Amphiphysin 2 (*BIN1*) [10,11], *RYR1* [12], *TTN* [13], *SPEG* [14], and *CCDC78* [15] (Table 1). Autosomal dominant mutations including Dynamin 2 (*DNM2*) were also identified, which linked this protein to membrane trafficking and cytoskeletal structure and dynamics [16].

Despite CNM’s genetic heterogeneity, the pathological consequences converge on common mechanisms. This review reframes CNM not merely as a membrane remodeling disorder, as previously suggested [17], but as a disease of impaired organelle communication and crosstalk. Specifically, this review will highlight how mutations in Myotubularin (*MTM1*) and Dynamin 2 (*DNM2*) compromise triad integrity and disrupt the physical and functional interactions between the sarcoplasmic reticulum (SR), mitochondria, and t-tubules. *MTM1* and *DNM2* are the most studied genetic drivers of CNM, accounting for most cases [18,19], and represent mechanistically complementary pathways. Their genetic and functional interactions provide the lens through which we will reframe CNM as a disorder of disrupted intracellular organization, with implications for cross-genotype therapeutic strategies. These defects propagate a cascade of cellular damage characterized by aberrant Ca^2+^ signaling, mitochondrial dysfunction, ROS dynamics dysregulation, and impaired autophagy. This interconnected model emphasizes CNM as a disease of organelle miscommunication, where a failure at one cellular region destabilizes others. This review provides the synthesis that targeting one CNM-associated pathway can mitigate dysfunction caused by another, providing compelling evidence for a converging organelle communication model and opening up new therapeutic strategies.

## 2. Genetic Heterogeneity and Major Genes at Play

### 2.1. MTM1

Myotubularin, a 3′-phosphoinositide phosphatase, is encoded by the *MTM1* gene. Its primary role is the management of lipid levels through the dephosphorylation of phosphatidylinositol 3-phosphate PI(3)P and phosphatidylinositol 3,5-bisphosphate PI(3,5)P2 into PI(5)P and phosphoinositide (PI) [20,21]. The purpose is to regulate various cellular processes, such as intracellular vesicular transport and endosome homeostasis. This is critical for cell signaling and membrane transport, excitation contraction coupling, intermediate filament organization, and neuromuscular junction structure maintenance [21,22].

*MTM1* loss-of-function mutation is the primary cause of X-linked recessive mutations, commonly referred to as XLMTM, and is the most common and severe form of CNM [23,24]. It disproportionately affects males at birth, leading to severe clinical features including hypotonia, muscle atrophy, overall weakness, difficulty breathing and swallowing, ptosis, ophthalmoplegia, and scoliosis [25]. Muscle biopsies feature severe size variation in fibers, with many small and round slow-twitch fibers and large hypertonic fast-twitch fibers, along with centralized nuclei and dysfunction in contractile machinery [26]. Furthermore, this mutation is often associated with a poor prognosis, with ~50-60% of patients dying within two years, primarily due to respiratory failure [27]. Additional non-muscle-associated phenotypes include acid reflux and constipation, sensory impairments, general cardiovascular involvement with specific congenital defects, a risk of liver hemorrhage, Cholestasis and hepatic peliosis, as well as respiratory infections [28,29,30,31].

Females with *MTM1* mutations were once thought to be asymptomatic. However, skewed X-chromosome inactivation has been associated with symptom presentation in females with muscle weakness [32]. Forty-three females carrying *MTM1* mutations had phenotypes ranging in severity from muscle weakness at birth to milder symptoms presenting in adulthood. Some also experienced breathing problems and facial movement issues consistent with male-associated phenotypes [33]. The clinical spectrum was expanded in 2023 in a report of a 23-year-old female patient with a heterozygous *MTM1* mutation, c.1261-10A>G, causing a premature stop codon. The milder XLMTM-associated phenotype presentation in this female patient compared to the typical presentation in males suggests partial expression of the *MTM1* gene, likely mitigating the severity. This underscores the importance of considering females with XLMTM and a need for improved diagnosis guidelines considering the phenotypic variability [34].

Clinically, *MTM1* has emerged as a therapeutic target, with some suggesting that myotubularin replacement or partial replacement can significantly improve or prevent this disease. Gene therapy utilizing adeno-associated viral vector AAV8 administration has been shown to rescue XLMTM cellular defects in murine and canine models [35,36]. A multinational, open-label, dose escalation study known the ASPIRO trial investigated the safety of resamirigene bilparvovec, an AAV8-based gene therapy. It was designed to deliver the *MTM1* gene to male patients under five years of age with XLMTM. The study achieved clinically significant improvements that diverged from the natural history of the disease, where patients typically remain ventilator-dependent and rarely achieve motor milestones. Data showed that 16 out of 17 (94%) patients achieved complete independence from ventilator support. Furthermore, patients reached unprecedented motor milestones including the ability to sit unassisted, stand without support, and, in some cases, walk independently. These functional gains were supported by biopsy data showing restored myotubularin expression and an increase in muscle fiber size. However, it cannot be said that all patients benefited. While the majority experienced functional recovery, the trial revealed a narrow therapeutic window and a significant safety threshold. Fatal adverse events took place for patients with underlying hepatobiliary disease, and the risks outweighed the potential for gene replacement. Thus, while the trial demonstrated potential for gene therapy for XLMTM, the benefit was not universal, and safety remains the primary barrier to broader application [37].

*MTM1* has been highlighted to intersect with other CNM pathways. Interestingly, the early systemic overexpression of *MTM1* has been shown as a promising avenue to mitigate BIN1-CNM in mice. This intervention showed improved muscle function and myofiber organization, showing normalized mitochondria positioning, corrected sarcomere organization, and rescued t-tubule biogenesis [38].

### 2.2. DNM2

The DNM gene family encodes for a family of dynamins. These are proteins with five structural domains, playing a role in endocytosis, exocytosis, intracellular membrane trafficking, and cytoskeleton organization through actin formation. *DNM2* is a ubiquitously expressed GTPase and is strongly associated with CNM [21,22,27,28]. In addition to CNM, *DNM2* mutations are most associated with the neurological CMT disease [39]; lethal congenital contracture syndrome 5, which leads to the failure of the neuromuscular system, resulting in death at birth [40]; and hereditary spastic paraplegia, causing adult-onset lower-limb spasticity [41].

*DNM2* has a broad range of functions in skeletal muscle. One of its most common roles is acting like a pair of molecular scissors, catalyzing membrane fission during clathrin-mediated endocytosis, creating small vesicles, as well as in exocytosis and vesicle trafficking. In CNM, the *DNM2* gain-of-function mutation becomes hypermorphic, causing the cell to treat t-tubules like vesicles, leading to fragmentation. Without intact t-tubules, the electrical signal is disrupted, leading to contractile dysfunction [42,43]. *DNM2* has also been identified to play an essential role in actin polymerization, as well as driving nuclei migration, spacing, and number maintenance. In CNM-linked mutation p.R465W, this process is disrupted, leading to a disorganized and weakened actin cytoskeleton. This causes problems such as GLUT4 accumulation defects, impairing the cell’s capacity to take up glucose efficiently. Furthermore, defects in nuclear position and number were observed [44,45]. Additional roles of *DNM2* are in recycling endosomes and autophagy. In CNM, *DNM2* cannot properly cut the membranes at recycling endosomes, preventing the cells’ formation of healthy autophagosomes [46].

*DNM2* expression levels are critically balanced, and overexpression or gain-of-function mutations are associated with autosomal dominant CNM, consistent with its pathological hallmarks of fiber atrophy, the centralization of nuclei, mitochondrial disruptions, sarcomere/triad disorganization, and reduced force. Interestingly, combining the opposing gain-of-function *DNM2* mutation of CNM and the loss-of-function *DNM2* mutation of CMT in mice resulted in functional compensation. This highlights the need to precisely normalize *DNM2* levels [47,48]. Therapeutically, this suggests that balancing *DNM2* expression levels is critical, rather than simply the suppression or enhancement of expression.

Various therapeutic strategies have been utilized to mitigate *DNM2*-associated CNM. Adeno-associated virus-expressing shRNA targeting the *DNM2* R465W mutation in young knock-in mice was utilized to target the gain-of-function mutation. This method exhibited phenotypic rescue for one year in the form of muscle mass restoration, fiber size normalization, and the decreased centralization of nuclei consistent with wild-type levels [49]. The versatility of this RNA interference approach has been further demonstrated in other disease variations. For instance, AAV-delivered shRNA was also employed to target the *DNM2* R369W mutation in mice. AAV9-shDNM2 was delivered through an intramuscular injection at 4 weeks. Treatment normalized *DNM2* to wild-type levels, leading to increased muscle mass and contractile force. The normalization also rescued histopathological hallmarks, including the decentralization of nuclei and restoration of t-tubule and mitochondrial abnormalities. Several limitations were observed, however, including incomplete mitochondria recovery and a lack of uniformity in rescue across muscle groups. This highlights that *DNM2* reduction must be precisely calibrated, further complicating the translational component [50]. A *DNM2* R465W knock-in mouse model was similarly targeted through AAV-delivered shRNA or antisense oligonucleotides. The treatment successfully rescued structural defects and corrected the fiber mass. Importantly, this study demonstrated that *DNM2* reduction could reverse the established muscle pathology in adult mice with pre-existing symptoms. While this treatment yielded significant improvements, challenges regarding lifelong dosing requirements and off-target effect potential in non-muscle tissue remain [51]. Combined, these studies suggest that *DNM2* downregulation has the potential to rescue CNM phenotypes. However, achieving similar efficacy in established adult human cases remains a significant challenge that requires further investigation.

The involvement of *DNM2* extends beyond a direct linear impact as it intersects with other CNM-associated pathways. This is evidenced through the targeting of *DNM2* to mitigate other CNM mutations. A single intramuscular injection of AAV-shRNA targeting *DNM2* in *MTM1* knockout mice achieved a stable ~50% reduction in protein levels, resulting in the comprehensive rescue of muscle mass and contractile strength. This intervention effectively normalized the fiber size and corrected XLMTM hallmarks including mitochondrial distribution and t-tubule network integrity [52]. Similarly, in *BIN1* knockout mice, targeting *DNM2* with antisense oligonucleotide downregulated its expression by 50%, restoring muscle function and mitochondrial organization, further highlighting the interaction of *DNM2* in *BIN1*-associated pathology [53].

## 3. Key Components of CNM

CNM is not caused by a single disrupted gene or pathway but rather a combined defective network. This network includes cell-structural defects in the nuclei and triad, Ca^2+^ dysregulation, mitochondrial dysfunction, ROS production imbalance, and the failure of homeostasis mechanisms to correct these errors. Mutations of CNM-associated genes trigger cellular defects, but the persistence of crosstalk between these pathways amplifies the pathological phenotypes, exacerbating disease presentation.

### 3.1. ROS and Mitochondrial Dysfunction in CNM

As early as 1985, Canal et al. reported, in a 34-year-old patient with CNM, the presence of mitochondria with paracrystalline inclusions near centralized nuclei, marking one of the earliest indications of the structural disruption of mitochondria in this disease. This suggested a link between organelle disruption and a hallmark of CNM—nuclei mislocalization [54]. In 1999, Naumann et al. provided further evidence of mitochondrial dysfunction as a consistent pathological feature of CNM [55]. Later studies have expanded on this observation, demonstrating that mitochondrial dysfunction, the overproduction of ROS, and defective mitophagy are recurrent features across the genetic variants of CNM.

Inherently, when maintained in balance, ROS serve several important physiological roles. Under normal conditions, skeletal muscle cells produce ROS during cycles of rest and contraction. At low levels, ROS are key secondary signaling molecules that are critical for proper skeletal muscle function and adaptation during exercise, calcium handling, mitochondrial biogenesis, and antioxidant defense [56,57]. ROS further contribute to transcriptional regulation as they can stimulate expression factors involved in redox regulation and mitochondrial dynamics, such as the PGC-1 α/β antioxidant defense mechanism [58]. Excessive ROS production tips the balance towards oxidative stress, which is associated with mitochondrial degradation, loss of muscle function, an increase in cell death, destabilization of Ca^2+^ homeostasis, and adverse impacts on contractility. Thus, the muscle’s capacity to adapt to injury and workload is supported by the beneficial response of ROS at lower levels [59,60].

The most common and severe form of CNM is XLMTM, caused by a mutation in the *MTM1* gene [61]. Recent work has expanded on the role of *MTM1* beyond membrane trafficking, identifying it as a regulator of organelle communication. *MTM1*-mediated PI(3)P hydrolysis is required for ER shape maintenance. The absence of *MTM1* leads the ER to undergo a sheet–tubule transition, further disrupting ER–mitochondria contact sites. This leads to a failure in mitochondrial Ca^2+^ uptake, causing energy defects and metabolic exhaustion associated with XLMTM [62]. Thus, these studies suggest that *MTM1* is critical for metabolic organelle coordination, aiding in cell adaptation under stress, and *MTM1* loss-of-function mutations result in a defective ER and an aberrant mitochondrial morphology, leading to a decline in ATP levels and disruption of cellular processes. While XLMTM patients experience disruption in different tissues, mitochondrial dysfunction seems to impact skeletal muscle through decreased mitochondrial respiratory chain enzyme activity, specifically without an impact on liver function [63]. Furthermore, centrally located nuclei and abnormal organelle clusters, including mitochondria mislocalization, as well as abnormal triads, disorganized sarcomeres, and neuromuscular junction enlargement, disrupting excitation–contraction coupling, contribute to impaired muscle contraction [64,65,66,67].

Later studies of mitochondria in a *DNM2* gain-of-function mutation CNM mouse model showed profound structural disruption, with swelling and a lack of cristae as the most severe structural defects. This observation was corroborated in patient muscle biopsies [66]. Most recently, a moderate mutation of *DNM2* in mouse models revealed similar disruptions to the mitochondria through enlarged and disrupted cristae structures. This suggests that even a less severe mutation leads to significant damage. Importantly, *DNM2*-associated defects were normalized after mutation correction, suggesting that gene therapy could be a significant therapeutic strategy for *DNM2* CNM reversal [50]. Complementary to these findings, muscle-specific *DNM2* knockout in mice demonstrated that *DNM2* is essential for lipid metabolism, mitochondrial function, muscle fiber structure, and neuromuscular junction integrity, as well as peripheral nerve function [68]. Dynamin 2 functions in the formation and maintenance of t-tubules and cell processes such as autophagy, lipid storage and regulation, mitochondrial energy production, actin formation and organization, clathrin-dependent and -independent endocytosis, and the support of glucose homeostasis through GLUT4 internalization [1,44,69].

Mitochondrial dysfunction in CNM is not limited to structural defects; they have a functional impact in disrupting Ca^2+^ handling and mitophagy, as well as severe impacts on neuromuscular junctions. These disruptions are tied to the triad, highlighting the functional interaction between t-tubules/ER and mitochondria [70,71]. This triad-focused dysfunction underscores that mitochondria do not act in isolation but instead rely on ER–t-tubule interactions. Specifically, mitochondria and the SR form mitochondria–SR contacts (MERCs), which are specialized microdomains that regulate Ca^2+^, energy metabolism, and redox signaling [72].

Together, these findings position *DNM2*- and *MTM1*-associated CNM as a disorder rooted in disorganized organelle communication and crosstalk. Specifically, the disruption of the ER–mitochondria connection leads to structural breakdown, impaired Ca^2+^ handling, and defective mitophagy, ultimately compromising the energy regulation of skeletal muscle. Interestingly, the early systemic overexpression of *MTM1* has been shown as a promising avenue to mitigate *BIN1*-CNM, improving muscle function and myofiber organization and showing normalized mitochondria positioning, corrected sarcomere organization, and rescued t-tubule biogenesis [38].

Evidence suggests that persistent mitochondrial dysfunction and excess ROS production in CNM likely do not act in isolation. Their alterations may carry significant disruptions in signaling pathways involving the nucleus and the ER/SR, potentially impairing the coordinated organelle network required for proper muscle homeostasis. A breakdown of mitochondrial miscommunication sets the stage for possible structural and functional defects observed in CNM.

### 3.2. Triad/T-Tubule Disorganization

The triad is composed of t-tubules, wedged between SR cisternae. Functioning as a specialized organelle mediating excitation–contraction coupling through Ca^2+^ release, it is a critical player in CNM disorders. It is also the site of dysfunction in a group of skeletal muscle disorders termed triadopathies, which encompass CNM and other congenital myopathies. In addition to its well-established role in contraction, it serves the role of a signaling hub for organelle crosstalk, thereby linking the plasma membrane, sarcoplasmic reticulum (SR), and mitochondria to coordinate Ca^2+^ dynamics, energy metabolism, and the architecture of the cell [72]. Disruption of this triad will lead to not only contractile dysfunction but also communication breakdown. This will lead to cellular distress in the form of mitochondrial dysfunction and a reduction in ATP production, defective autophagy, and increased ROS production [73]. Emerging evidence reveals an additional layer of ROS amplification. Work by Neitzel et al. showed that loss of *MTM1* and *BIN1* disrupts the t-tubule architecture, leading to the compromised compartmentalization of extracellular protons [74]. This has the potential to impact ion channel function, and consequently, Ca^2+^ dynamics in muscle fibers, contributing further to muscle fiber dysfunction. Furthermore, extracellular acidification has been linked to ROS tolerance [75,76].

Following the understanding of the triad as a central signaling hub, a key pathological feature of CNM is triad and t-tubule disorganization. XLMTM mutation leads to disruptions in triad and t-tubule structures. Direct in vivo evidence of *MTM1* knockout in zebrafish recapitulates the XLMTM hallmarks, revealing misaligned or missing t-tubule/SR junctions. These findings suggest that myotubularin plays a critical role in early t-tubule formation, and defects lead to long-term breakdown in excitation–contraction coupling [67]. In line with this, through the regulation of PtdIns3P by myotubularin, the remodeling of the SR takes place in vivo, providing direct evidence that *MTM1* loss impacts SR organization and thus causes the destabilization of the triad [77]. Complementary to these findings, Cowling et al. reported that the overexpression of *DNM2* in adult skeletal muscle cells leads to significant triad disorganization [48]. Further supporting the central role of membrane remodeling in triad formation and maintenance, Fujise et al. demonstrated that CNM mutations in *DNM2* and *BIN1* disrupt membrane tubulation, impair t-tubule biogenesis, and lead to severe disorganization in triad structure. This work highlights how an imbalance in *DNM2* activity contributes to excitation–contraction coupling failure through fragmented t-tubules and disorganized triads [78].

The convergence of these defects highlights a shared pathologic mechanism across CNM types, particularly *MTM1* and *DNM2*, along with *BIN1*. In these cases, foundational errors in membrane remodeling and organelle positioning ultimately impair the efficiency of excitation–contraction coupling [61]. These triad abnormalities initiate a pathological feedback loop: impaired calcium release leads to chronic cell stress, further driving mitochondrial dysfunction, ROS accumulation, and impaired autophagy. Consequently, the failure of these systems further drives the destabilization of the triad and myofibers themselves.

Together, these studies provide mechanistic insights into the underlying muscular weakness characteristic of CNM, supporting a model in which the disease is not merely a static structural disorder. Instead, the data point towards a dynamic loop of dysfunction that may amplify the initial pathological impact. Triad and t-tubule disorganization in CNM can fundamentally compromise the physical and functional dialog between the sarcolemma and SR. This disruption weakens the mitochondrial Ca^2+^ buffering capacity, suggesting that the loss of this precise spatial crosstalk can transform a localized membrane defect into widespread organelle dyscoordination. Ultimately, such breakdown may serve to amplify the downstream pathology, creating a self-sustaining cycle of muscle impairment. This paradigm may provide new points of entry for novel therapeutics.

### 3.3. Nuclear Mispositioning and Cytoskeletal Instability

Nuclear positioning is critical for proper muscle function. During normal skeletal muscle maturation, after myoblast fusion, the newly formed myonuclei initially cluster at the center of the nascent myotube. Subsequently, these nuclei undergo a relocation process to become peripherally arranged along the edge of the muscle fiber. The initial centering of myonuclei during fusion is orchestrated by microtubules and motor proteins—specifically the dynein/dynactin complex. These components move along the microtubule network and are coordinated by organizing centers—most notably the centrosome—during early muscle differentiation [79,80]. Adding to this complex architecture, centrosome-organized microtubules and the LINC (Linker of Nucleoskeleton and Cytoskeleton) complex play central roles in nuclear positioning. Following the centralization of nuclei after myoblast fusion as directed by centrosome-organized microtubules, the LINC component Nespirin-1a plays a crucial role by recruiting centrosome pericentriolar material (PCM) to the nuclear envelope. This process triggers a “relocalization” program termed centrosome reduction, whereby centrosome proteins reorganize around the nuclear envelope, transforming it into a non-centrosomal microtubule-organizing center (ncMTOC) [80,81,82]. Centrally located nuclei are a hallmark of CNM and an indicator of significant pathology and disruptions as they impact gene expression, structural stability, and the overall force conduction of the muscle [83]. Thus, the loss of Nespirin-1a or associated components likely leads to an impairment in centrosome reduction, potentially leading to microtubule disorganization. This can lead to further disruptions of proper organelle positioning and could be an underlying cause of the nuclear mispositioning phenotypes observed in CNM.

During later stages of myofiber maturation, peripheral nuclear positioning is driven in part by the intermediate filament desmin, which crosslinks sarcomeres; combined with myofiber contraction, this network pushes nuclei towards the periphery. They are subsequently anchored by nuclear envelope proteins and stabilized through Lamin A/C-regulated nuclear stiffness. The proper peripheral positioning of nuclei requires an orchestrated interplay among these cytoskeletal and nuclear envelope-associated systems. A complex of Arp2/3, Arpc5L, and gamma actin is needed to arrange desmin, which links the myofibrils, allowing nuclei to move [84] Additionally, *DNM2* mutations uncouple Dynamin 2 from actin remodeling, leading to significant trafficking defects, cytoskeletal instability, and structural disorganization in the fiber [44,84].

While Cadot et al. focused on the mechanics of nuclear positioning, their findings provide a foundation for a more expansive disease model. In this view, nuclear mispositioning and membrane defects act alongside dysfunctional organelle crosstalk to create a self-reinforcing cycle common to various CNM genotypes. Collectively, these defects form a maladaptive loop that underlies progressive muscle dysfunction.

In support of this integrative view, Hnia et al. demonstrated that loss of *MTM1* disrupts the desmin intermediate filament (IF) network. In *MTM1* knockout mice and XLMTM patient biopsies, desmin IFs form uncharacteristic aggregates in the perinuclear region, instead of an organized lattice. The lack of radial patterns disrupts nuclei anchoring, leading to nuclear clustering. They also found mitochondria to be disorganized, particularly around regions of desmin aggregation. This suggests that perinuclear desmin IFs are important for both nuclear and mitochondrial positioning [65]. Excess *DNM2* in a mouse knock-in model demonstrated that protein overabundance contributes to the destabilization of the cytoskeleton, leading to desmin accumulation, triad stress, and progressive nuclear mispositioning. Successfully rescuing this phenotype restored structural integrity to the muscle tissue [50]. This highlights the central role of *MTM1* and *DNM2* in maintaining the structural integrity of the perinuclear cytoskeleton through desmin IF organization, Ca^2+^ regulation, and organelle organization. This links the collapse of the perinuclear structure to the overall XLMTM pathology. Additional evidence in support of the importance of *MTM1* stability in nuclear positioning comes from the work of Gupta et al. They demonstrated that loss of lipid phosphatase MTMR12 in zebrafish led to *MTM1* destabilization. This destabilization resulted in centralized nuclei and CNM pathology. This work highlights the crucial nature of the cytoskeletal structure and nuclear positioning preservation [85].

Mispositioned nuclei and an unstable cytoskeletal network in CNM are reflective of the profound failure of nucleocytoskeletal coupling, which normally relies on a continuous cycle of signaling alongside the mitochondria, ER, and plasma membrane. When this communication network collapses, the entire spatial organization of the muscle fiber is compromised, likely preventing other organelles from maintaining their proper positioning and function.

### 3.4. Calcium Dysregulation

Dysfunction in triads and t-tubules is often coupled with Ca^2+^ handling dynamics, and this is consistent across the CNM subtypes. The foundational work by Fraysse et al. directly linked calcium handling defects to *DNM2*-CNM. In *DNM2*-overexpressing mice, impaired excitation–contraction coupling (ECC) occurred through defective Ca^2+^ release from the SR despite preserved Ca^2+^ uptake, pointing to a plasma membrane origin [86]. Subsequently, physiological evidence in *DNM2* knock-in mice confirmed the structural disorganization, misalignment, and reduced density of triads and SR/t-tubules. This was accompanied by a reduction in Ca^2+^ transients in response to depolarization originating from a compromised signaling cascade with no impact on the Ca^2+^ load, further implicating structural defects [48,86].

Parallel studies focusing on *MTM1* further demonstrate this convergence of structural defects in the triad with Ca^2+^ handling. *MTM1* loss leads to disrupted t-tubule networks, disorganized Ca^2+^ signaling, and depressed voltage-activated SR Ca^2+^ release (~60% reduction) [87]. In healthy mammalian muscle fibers, RyR1-mediated Ca^2+^ release from the SR is strictly regulated by the voltage-sensing CaV1.1 channel in the t-tubules to prevent spontaneous Ca^2+^-induced Ca^2+^ release. Notably, myotubularin deficiency alters RyR1 gating, shifting the receptor towards an abnormal state. This triggers prolonged Ca^2+^ release events that occur independently of physiological voltage gating. Myotubularin is suggested to “unlock” this abnormal mode of Ca^2+^ release, causing spontaneous and prolonged Ca^2+^ release events [88,89].

A rare CNM-linked mutation is a biallelic autosomal recessive mutation in the striated muscle-enriched protein kinase encoded by the SPEG gene. A zebrafish knockout model of this gene highlights the CNM hallmark of the disorganization of triads and defective t-tubules. Furthermore, Ca^2+^ imaging of muscle fibers indicates significantly reduced Ca^2+^ transients, a consistent feature of impaired excitation–contraction coupling [90]. Collectively, these findings across *DNM2*-, *MTM1*-, *BIN1*-, and *SPEG*-related CNM illustrate a self-enforcing pathological loop. In this model, genetic defects disrupt the triad/t-tubule architecture, leading to altered Ca^2+^ signaling and ECC, resulting in progressive muscle weakness, and emerging sensors such as GPR68 offer a new therapeutic entry point.

Ca^2+^ mishandling in CNM arises not only from defective RyR1/DHPR channels but also from disrupted physical and molecular bridges that link the SR, mitochondria, and t-tubules. The disruption of this interorganelle Ca^2+^ dialog creates a loop of ER/SR distress, mitochondrial overload, and failure of proper contractile function. This positions Ca^2+^ dysregulation as both a consequence and perpetuator of failed organelle crosstalk.

### 3.5. Impaired Autophagy and Mitophagy

Autophagy is a conserved degradation pathway that is critical for the removal of damaged organelles and misfolded proteins through lysosomal degradation. This process is especially critical in metabolically active cells such as skeletal muscle cells [91]. A recent review by Xia et al. highlights that the precise regulation of autophagy is essential for muscle homeostasis. A deficiency or excess of autophagic activity can lead to a variety of myopathies. Furthermore, it emphasizes the intricate links to various key singling pathways, like mTOR, rather than being an isolated process [92]. A failure at any stage of the autophagy pathway, including induction, cardio recognition, autophagosome formation, or lysosomal degradation, serves as an underlying mechanism for various disorders. These include neurodevelopmental and neurodegenerative disorders, as well as genetic skeletal myopathies and congenital myopathies such as CNM [93]. In CNM, impaired autophagy contributes to mitochondrial dysfunction and organelle mislocalization [94,95]. Abnormal autophagy has been a common feature across several genetic forms of CNM, including *MTM1*, *DNM2*, and *BIN1*. Overall, these genes are regulators of membrane trafficking and phosphoinositide signaling, which directly intersect with autophagy pathways [17]. One specific type of autophagy is mitophagy, responsible for damaged mitochondria elimination. Complementary to autophagy studies, recent work in mitochondrial myopathies suggests a “mosaic mitophagy response” whereby mitophagy is elevated near central nuclei in early fibers only to be blocked in advanced “ragged-red fibers” containing abnormal mitochondria, leading to the accumulation of LC3/P62 markers. This accumulation is linked to mTORC1 overactivation, leading to a block in degradation pathways. This was found to be reversible through rapamycin treatment, boosting mitophagy [96].

Supporting this idea, *DNM2* mutant mice showed the accumulation of autophagic markers (LC3-II and p63), indicating a block in autophagy progression. This defect in autophagy coincided with disorganized triads and the abnormal localization of organelles and mitochondria, expanding *DNM2*’*s* role beyond endocytosis and membrane trafficking to autophagy and autophagosome maturation [97]. Furthermore, recent work shows that *DNM2* drives the scission of nascent autophagosome precursors through LC3-dependent interaction, which is critical for autophagosome formation. A defect in *DNM2* prevents this from taking place, causing immature autophagosome accumulation [46]. Similarly, in *MTM1*-deficient mice, muscle hypertrophy emerged alongside autophagy perturbation marked by autophagosome accumulation and a rise in LC3/P62 markers. This suggests a mismatch between autophagosome formation and clearance. The correction of MTM deficiency through AAV-mediated *MTM1* delivery normalizes the muscle mass, as well as LC3/P62 levels, suggesting that the *MTM1* pathway modulates autophagy [73]. This highlights that *DNM2* and *MTM1* mutations disrupt proper autophagy, underscoring its failure as a core driver of CNM pathology, rather than a secondary consequence. Altogether, these studies support a model where defective autophagy/mitophagy is a point of convergence for CNM pathologies, linking membrane remodeling defects to disintegrating organelle quality control. Furthermore, this suggests that the modulation of autophagy/mitophagy regulators (e.g., mTORC1) through activators of autophagy (e.g., rapamycin) may enabling the therapeutic rebalancing of organelle crosstalk in CNM.

Defective autophagy and mitophagy in CNM are indicators of the terminal breakdown of organelle communication networks, which depend on intact signaling between lysosomes, mitochondria, and the nucleus. When mitochondria cannot signal their damage, lysosomes cannot reach them, and the nucleus cannot launch the appropriate transcriptional response. Thus, damaged components accumulate, setting the fate of the myofiber. Thus, impaired organelle clearance can be viewed as a manifestation of lost organelle communication in CNM.

## 4. Integration: A Unified Pathological Loop

Mutations in *MTM1* and *DNM2* converge on the shared failure of membrane remodeling, which destabilizes the triad and disrupts organelle communication in CNM. Despite acting at different molecular points, both mutations impair the t-tubule structure, alter calcium signaling, and trigger mitochondrial dysfunction and defective autophagy, collectively initiating the broader pathological loop. In this context, the central nuclei represent more than just a structural abnormality; they are the visible sign of a fundamental breakdown in how cellular compartments communicate. When the nucleus cannot maintain its proper position, this reflects widespread problems in the cell’s cytoskeleton and metabolism, making it both a diagnostic marker and an active contributor to disease progression. Importantly, therapies that restore communication between organelles, whether through gene replacement, *DNM2* normalization, or metabolic interventions, can correct nuclear positioning, proving that this hallmark feature is reversible.

This organelle miscommunication creates a self-amplifying pathological cycle where defects in one compartment cascade through the entire cellular network, with each failure exacerbating the others. The cycle unfolds as follows.

- Initiation: Mutations in *MTM1*, *DNM2*, *BIN1*, or *SPEG* disrupt the triad architecture and membrane remodeling machinery.
- Signal disruption: These structural defects impair excitation–contraction coupling, reducing the calcium transient amplitude and causing asynchronous calcium release.
- Metabolic crisis: Isolated mitochondria cannot properly respond to calcium signals, leading to ATP deficiency and excessive ROS production.
- Quality control failure: Disrupted autophagy prevents the clearance of damaged organelles, allowing ROS and misfolded proteins to accumulate.
- Structural degeneration: Cytoskeletal instability and energy deficits lead to nuclear mispositioning, sarcomere disorganization, and progressive muscle weakness.
- Amplification: Each step feeds back to worsen the others—ROS damages membranes and further impairs calcium handling, ATP loss prevents autophagy, and cytoskeletal defects worsen triad disorganization.

**Figure 1:**
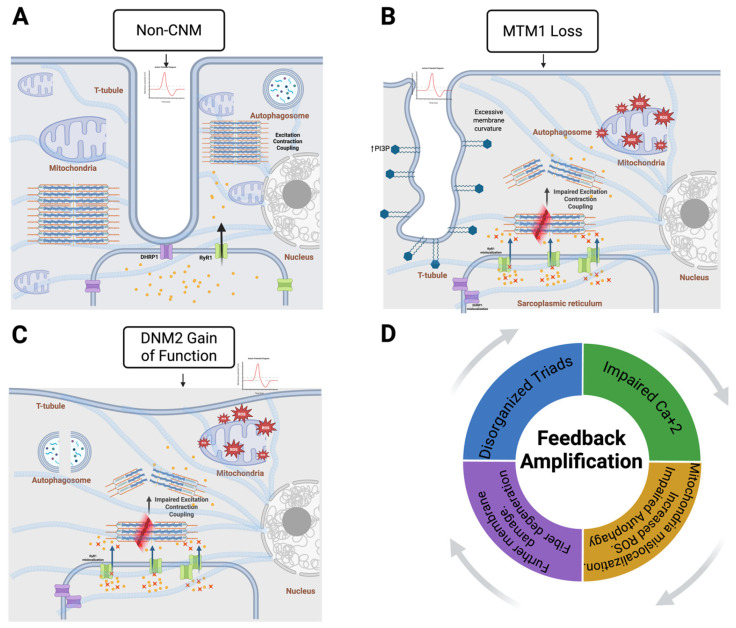
Centronuclear myopathies present disorganized triads, autophagy, and mitochondrial stress. (**A**) In non-CNM skeletal muscle fibers, properly organized t-tubules and the sarcoplasmic reticulum form intact triads and support normal excitation–contraction coupling, balanced calcium handling, mitochondrial function, and regulated autophagy. (**B**) In *MTM1*-associated CNM, excessive membrane curvature and phosphoinositide dysregulation lead to triad disorganization, impaired excitation–contraction coupling, mitochondrial dysfunction, and the accumulation of autophagosomes. (**C**) In *DNM2* gain of function-associated CNM, increased membrane fission and altered cytoskeletal dynamics, a disrupted triad architecture, and defective calcium handling occur, promoting mitochondrial stress and autophagy imbalance. (**D**) These defects converge into a pathogenic feedback loop in which disorganized triads impar calcium homeostasis, driving mitochondrial dysfunction and elevations in ROS production, further exacerbating autophagy dysfunction and muscle fiber disorganization.

This dynamic loop explains why CNM is not a static structural disorder but rather a progressive condition where early interventions can break the cycle and halt disease progression (Figure 1).

## 5. Key Outstanding Questions

Despite advances in our understanding of CNM, several key questions remain unanswered, including the determinants of disease onset, fiber type susceptibility, and how lessons from other forms of muscle degeneration can be applied to CNM. This section explores these gaps in knowledge and proposes integrative avenues for investigation.

The variable timing of disease onset among CNM subtypes suggests a fundamental difference in developmental timing and compensatory mechanism activation. One potential explanation for this variability lies in the diversity of the affected proteins, as well as the necessity of this role in cell function. Myotubularin, for example, encoded by *MTM1*, is critical for t-tubule function and membrane remodeling and is required for fetal myogenesis. Its absence causes immediate structural collapse. Typically, congenital cases with onset in males carry a mean age of death of ~7 years. Female carriers present with more variable onset, with a mean of 17 years, ranging from congenital to adult. In contrast, *DNM2* mutations may subtly impair cytoskeletal remodeling and trafficking, consequently leading to progressive stress and damage during postnatal growth. Recent cohort studies highlight this phenotypic spectrum emphasizing the importance to consider this disease in older age diagnoses [98]. Onset is usually in childhood or adolescence, with a range of 1 to 46 years [19,98,99]. Future studies using patient-derived induced pluripotent stem cells (iPSCs), including those generated from individuals with CNM, could help to identify molecular switches that distinguish early- versus late-onset disease phenotypes [99].

Skeletal muscle fibers exhibit distinct metabolic and contractile properties, and their unique vulnerabilities may modulate disease progression in CNM. For instance, type I (slow-twitch, oxidative) fibers are mitochondria-rich and fatigue-resistant and are often preferentially affected in *MTM1* and *DNM2* models [11,100]. While the mechanism behind this is not fully understood, the higher mitochondrial load and increased dependence on excitation–contraction coupling could provide an explanation [101]. These factors render these fibers more sensitive to organelle dysfunction and more vulnerable to t-tubule and membrane disruptions. Emerging tools like fiber-specific RNA sequencing, laser capture microdissections, and spatial proteomics could help to unravel the molecular underpinnings of fiber sensitivity and selectivity in CNM pathology [102,103,104].

Although CNM is a genetic disorder, it possesses shared features with age-related muscle decline. Insights from other forms of muscle decline, particularly aging and muscular dystrophy, may provide a conceptual framework within which to understand CNM progression. Age-related sarcopenia is increasingly recognized to be driven by cellular senescence. Targeting of senescent cells has shown therapeutic potential to improve muscle health in aging populations [105]. Studies of dystrophic muscle environments reveal senescence implications as well. Specifically, senescent fibro-adipogenic progenitors (FAPs) were shown to accumulate and impair muscle regeneration. Senolytic treatment with fisetin reduces senescent FAPs and restores muscle progenitor function [106]. These studies suggest that senescence may contribute to disease progression in CNM, specifically in later-onset forms.

Exercise biology further underscores the complexity of muscle adaptation and degeneration and could offer additional insights into CNM. In Duchenne muscular dystrophy (DMD), exercise can exert a beneficial and adverse effect depending on the intensity and context. The activation of SIRT1 with SRT2104 exerts exercise-mimetic effects, promoting recovery in DMD models and thus mimicking the benefits of physical activity [107]. Conversely, downhill running in *mdx* mice increased endomysial fibrosis and oxidative DNA damage while reducing the myonecrosis area, showing that exercise can have complex, sometimes detrimental effects in dystrophic muscle [88]. Exercise was suggested to counteract age-related senescent cell accumulation by activating immune responses and addressing DNA damage, mitochondrial dysfunction, and inflammation [108]. In aging mice, aerobic exercise training induced mitonuclear imbalance and the activation of the mitochondrial unfolded protein response (UPRmt), potentially mitigating age-related mitochondrial decline [93], while also rejuvenating quiescent skeletal muscle stem cells in old mice by restoring cyclin D1, enhancing the regenerative capacity [109]. These studies suggest that exercise could be leveraged to improve mitochondrial health and stem cell function in CNM, with the consideration that inappropriate mechanical stress may exacerbate underlying structural fragility.

Finally, emerging evidence highlights the importance of mitochondrial–satellite cell crosstalk in muscle maintenance and repair. The restoration of mitochondrial function has been shown to support muscle satellite cells (MSCs) in repairing after injury, reduce fibrosis, and improve regeneration following injury [110]. Given that mitochondrial dysfunction, mislocalization, and impaired quality control are features of CNM, targeting mitochondrial health may present a viable therapeutic strategy capable of enhancing the regenerative capacity even in the presence of genetic defects.

Together, these observations suggest that CNM should be viewed as a disease shaped by developmental timing, fiber type vulnerability, organelle dysfunction, and an evolving niche. Integrating insights obtained from aging, dystrophy, and exercise studies may help to uncover modifiers and potential intervention points capable of modulating CNM subtypes and increasing resilience.

## 6. Therapeutic Approaches and Limitations

Efforts to break the maladaptive loop of dysfunction in CNM have led to the development of diverse therapeutic strategies. However, their success remains limited constrained by a narrow therapeutic window and translational challenges.

The translational trajectory of CNM therapies has been heavily informed by animal models. Notably, the canine model of myotubularin deficiency demonstrated that systemic AAV8-mediated gene therapy can drive whole-body correction and long-term survival in dogs [36]. The downside of this model is that it does not predict the dose-dependent hepatoxicity encountered in human patients. Recombinant AAV gene therapy was assessed for its ability to normalize the myofilament and myonuclei mechanics of the muscle fibers. Early intervention suggested the restoration of contractile force in *MTM1*-deficient mice, as well as correcting the myonuclear volume, scaling, and localization along the cell. Although rAAV therapy has been shown to be capable of phenotype adjustment at a cellular level, the primary challenge is the translational limitation. Bridging the gap between animal models and human physiology remains the primary bottleneck. Achieving therapeutic cellular concentrations systemically without triggering dose-limiting toxicities remains an unresolved challenge [111]. These discrepancies underscore the challenges of using animal models to establish safe margins to carry into clinical trials. In translating such preclinical benchmarks into human subjects, the ASPIRO trial remains the primary benchmark for these efforts. As previously detailed, however, it also remains a cautionary tale regarding the safety of high-dose systemic AAV delivery [37].

An alternative strategy has emerged that focuses on modulating existing genetic modifiers to overcome the safety constraints surrounding gene replacement therapies. Utilizing antisense oligonucleotides, a type of drug-like molecule, this study provides biological support for the idea of “cross-therapy”, where targeting one gene can compensate for a mutation in another. *DNM2* reduction extended the lifespan of the mice and restored muscle force and histology in a dose-dependent manner. Critically, this study highlights the importance of early intervention and precise dose titration, indicating a translational hurdle that must be overcome. An additional challenge is maintaining the levels of the ubiquitously expressed *DNM2* within a functional physiological range [112]. The versatility of *DNM2* reduction as cross-genotype strategy was further extended io SPEG-deficient CNM. The study demonstrated that *DNM2* reduction can normalize the triad number and ultrastructure, thereby restoring Ca^2+^ handling. Crucially, *DNM2* reduction failed to rescue the cardiac phenotype in *SPEG*-deficient models. This suggests that *DNM2* modulation may only serve as a skeletal muscle-specific component of a more comprehensive, multi-organ therapeutic strategy [113].

Genetic interventions carry delivery and safety complexities. To address this, pharmacological repurposing has been explored to target the downstream signaling pathways of CNM. An example of this approach is the use of tamoxifen, which utilizes estrogen receptor signaling to reduce *DNM2* levels, providing the potent, small-molecule rescue of muscle function. The utilization of an FDA-approved drug provides the advantage of an immediate preclinical foundation. However, high doses of tamoxifen reduced food intake, leading to weight loss in mice, a side effect requiring monitoring in human populations. On the other hand, low-dose tamoxifen, consistent with human pediatric doses, did not fully reverse the central nuclear positioning, suggesting that structural adjustment requires a high dose [114]. A promising therapeutic avenue to counteract the loss of *MTM1* phosphatase activity involves the reciprocal inhibition of phosphoinositide-synthesizing kinases. PIK3C2B was established as a potent genetic modifier, where its ablation could completely prevent or reverse the MTM phenotype by restoring PI3P homeostasis and triad integrity. While the inhibition of PIK3C2B is a promising therapeutic strategy, inhibition of the related PIK3C3 kinase is detrimental and can exacerbate the disease through autophagic dysfunction. Another significant challenge to this approach is the lack of selective PIK3C2B inhibitors, preventing a transition to human clinical trials [115].

In contrast to previous approaches, Lawlor et al. demonstrated the viability of a targeted enzyme replacement agent, the 3E10Fv-*MTM1* fusion protein. The aim of this proof of concept was to show that exogenous myotubularin can penetrate the sarcolemma to restore the triads, addressing the excitation–contraction coupling failure driving weakness in XLMTM. However, this study highlighted a translational gap, where, even though functional and ultrastructural gains were rapid, histopathological markers like nuclear positioning remained unchanged. This suggests that this intervention method requires long-term administration to achieve phenotypic rescue [116]. The limitations of a single treatment approach are critical to address. Given the highlighted challenges, the future of CNM therapeutics likely lies in an integrative strategy that combines functional rescue through pharmacological intervention or protein replacement alongside long-term genetic modulation. A comprehensive strategy can achieve structural normalization and systemic safety.

While definitive disease-modifying therapies are currently in development, established management strategies focus on mitigating the clinical progression of congenital myopathies. As outlined in the international consensus statement, these disorders require a robust multidisciplinary approach to address the consequences of organelle dysfunction and multi-tissue involvement [117]. The mitigation of physical decline in congenital myopathies relies on a balanced exercise regimen. High-intensity aerobic exercise was not associated with significant muscle damage or pathological elevation in creatine kinase in a mixed group of patients representing various myopathies [118]. Similarly, progressive resistance training has been shown to increase maximal voluntary contraction and functional walking capacity, suggesting that this can be an effective way to manage disease symptoms [119]. The pharmacological mitigation of organelle failure often involves a “mitochondrial cocktail” designed to stabilize ATP production. Additionally, this method offers ROS neutralization aimed at protecting the SR from secondary oxidative damage, thereby maintaining muscle viability during periods of physiological stress [120]. Addressing the respiratory muscle weakness is a critical component in disease mitigation. Park et al. demonstrated a correlation between reduced muscle strength and impaired cough capacity as an indicator of respiratory function. Management of this deficit with techniques like assisted coughing and non-invasive ventilation is essential to prevent further physiological complications observed in CNM and other congenital myopathies [121]. In totality, these mitigation methods are aimed at enhancing patient survival and quality of life.

## 7. Conclusions

In conclusion, CNM is a complex disease driven by a heterogenous set of genetic mutations such as *MTM1* and *DNM2*. This review has demonstrated that these defects manifest as the fundamental disorganization of organelle communication and crosstalk. The initial genetic insult triggers a cascade of structural and functional pathologies, ranging from cytoskeletal instability to disorganized triads, leading to aberrant Ca^2+^ signaling and impaired mitochondrial function. This culminates in chronic cell stress and a vicious cycle of dysfunction, where each failure has the capacity to exacerbate others. This ultimately renders the skeletal muscle fiber unable to carry out its functions. An in-depth and holistic understanding of this dynamic loop of organelles and the interconnected pathways is essential for developing targeted therapies to mitigate this disease. Early successes with gene therapy trials aimed at replacing defective genes indicate a promising approach. Through the correction of the initial defect, this has the potential to break the cycle of organelle miscommunication and restore cell function, thus reversing the defects of this congenital disease. Finally, given the rare nature of centronuclear myopathies, there remains a critical need for large-scale patient registries across global natural history. Such data will be essential in identifying population-specific phenotypes and determining whether demographic variations influence the underlying mechanisms of organelle crosstalk and disease progression.

## Figures and Tables

**Table 1:** Genetic causes of centronuclear myopathies and associated molecular features and clinical severity. This table summarizes key genes implicated in centronuclear myopathies, their inheritance patterns, the key molecular functions of each gene, characteristic defect patterns, and the relative clinical severity. Collectively, these genes converge on pathways that regulate membrane remodeling, cytoskeletal organization, excitation–contraction coupling, and organelle positioning, highlighting a shared mechanism underlying muscle fiber disorganization and disease heterogeneity.

Gene	Inheritance Pattern	Key Molecular Function	Hallmark Defects	Clinical Severity
*MTM1*	X-Linked Recessive	Lipid phosphatase, hydrolyzes PI3P to maintain membrane homeostasis	T-tubule disorganization, impaired calcium handling, central nuclei, and mitochondrial mispositioning	Severe (XLMTM often neonatal onset)
*DNM2*	Autosomal Dominant	GTPase involved in membrane cleavage and actin cytoskeleton remodeling	Actin cytoskeleton defects, nuclear clustering, impaired trafficking, and mild t-tubule defects	Mild to moderate, later onset
*BIN1*	Autosomal Recessive/Dominant	Membrane curvature sensing, t-tubule biogenesis	Disrupted t-tubule formation, defective excitation–contraction coupling, and central nuclei	Variable, some neonatal cases
*RYR1*	Autosomal Dominant/Recessive	Calcium release channel of sarcoplasmic reticulum	Impaired excitation–contraction coupling, fiber type susceptibility, and mitochondrial stress	Variable, overlaps with other myopathies
*TTN*	Autosomal Dominant/Recessive	Sarcomere assembly- giant structural protein (Titin)	Sarcomere disorganization and central nuclei	Variable, late onset
*SPEG*	Autosomal Recessive	Kinase involved in triad and membrane dynamics	Triad fragmentation, altered calcium signaling, and mitochondrial defects	Moderate to severe

## Data Availability

No new data were generated or analyzed in this study.

## Abbreviations

The following abbreviations are used in this manuscript:

CNM	Centronuclear Myopathy
NMD	Neuromuscular Disease
ROS	Reactive Oxygen Species
ER	Endoplasmic Reticulum
XLMTM	X-Linked Myotubular Myopathy
*DNM2*	Dynamin 2
*MTM1*	Myotubularin 1
PI3P	3′-Phosphoinositide Phosphate
PI	Phosphatidylinositol
SR	Sarcoplasmic Reticulum
CMT	Charcot–Marie–Tooth
LINC	Linker of Nucleoskeleton and Cytoskeleton
PCM	Pericentriolar Material
ncMTOC	Non-Centrosomal Microtubule-Organizing Center
IF	Intermediate Filament
ECC	Excitation–Contraction Coupling
RyR1	Ryanodine Receptor
FAP	Fibro-Adipogenic Progenitor
UPRmt	Mitochondrial Unfolded Protein Response
MSCs	Muscle Satellite Cells
